# Occupational stress and body composition of hospital workers: a follow-up study

**DOI:** 10.3389/fpubh.2024.1459809

**Published:** 2024-10-17

**Authors:** Carlos Rodrigo Nascimento de Lira, Rita de Cássia Coelho de Almeida Akutsu, Lorene Gonçalves Coelho, Renata Puppin Zandonadi, Priscila Ribas de Farias Costa

**Affiliations:** ^1^School of Nutrition, Federal University of Bahia, Salvador, Brazil; ^2^Department of Nutrition, Campus Darcy Ribeiro, University of Brasilia, Brasília, Brazil; ^3^Health Science Centre, Federal University of Recôncavo of Bahia, Santo Antônio de Jesus, Brazil

**Keywords:** linear models, worker’s health, likelihood functions, anthropometry, mixed effects models, cohort study

## Abstract

This study sought to analyze the influence of occupational stress on the body composition of hospital workers after one year of follow-up. This prospective cohort study included 218 workers from one of the leading private hospitals in the municipality of Santo Antônio de Jesus, Recôncavo da Bahia region, Northeast Brazil. Body composition was analyzed by proxy (Body Mass Index and Waist Circumference) and Bioelectrical Impedance Analysis. The primary exposure adopted in the present study was the perception of occupational stress, assessed with the adapted and reduced version of the Job Content Questionnaire evaluating demand and control dimensions. The covariates were work characteristics; biological characteristics; sociodemographic characteristics and lifestyle. Statistical analyses were performed using descriptive, bivariate and multivariate analysis. At the first stage of the study, we identified that 55.96% (*n* = 122) of workers had high work demand and 25.22% (*n* = 55) had low control. Among those who had high demand and low control at the beginning of the study, the majority were women, non-white, with low educational and income levels, sleeping less than 7 h/day. After 12 months of follow-up, the median value for demand continued as 13 (IQR: 5–25) and for control, it increased to 16 (IQR: 9–23). In this second moment of the study, 62.38% (*n* = 136) of workers showed high demand and 45.87% (*n* = 100) low control. The characteristics of workers with high demand and low control were similar to those of the first moment. The results indicate that high demand and low control at work are risk factors for changes in body mass index, fat mass and fat-free mass in hospital workers. This study shows the importance and need for clinical and epidemiological assessments regarding the body composition of professionals working in hospitals, since high rates of overweight and obesity are triggers of chronic health problems such as dyslipidemia, diabetes mellitus and cardiovascular diseases, among others. Therefore, managers must promote adequate working conditions and understand the need for periodic body composition assessments.

## 1 Introduction

Occupational stress is present when stressful factors are related to the work environment and workers do not have the capacity or resources to adapt to such factors (1). Stress is considered a public health problem affecting different individuals and is present in several organizations, including hospitals (1–3). Evidence point to a high prevalence of occupational stress among hospital workers, ranging from 23.8 to 87% (4–7), reinforcing the need for studies and interventions in this work environment.

Not neglecting occupational stress and talking about it is essential and urgent, especially given the precariousness of work environments (8, 9). Understanding that adequate working conditions constitute a human right and, given millions of people worldwide suffering from the consequences of precarious work, an attentive and dedicated look from managers and an approach that reduces occupational stress are priorities, as they contribute to physical and mental health.

Several theoretical models regarding occupational stress are observed in the literature, with the Demand-Control Model being a reference model and one of the most used in the world. The work psychosocial dimension in this model is Job Demand, which refers to the psychological stressors involved in carrying out work, such as unexpected tasks, the volume of work, time pressures, level of attention and concentration required, etc. (10–12). The Control dimension concerns to the control that the worker has when carrying out his tasks and the conduct adopted during the working day, such as autonomy, use and development of skills in carrying out tasks, initiative, and influence on organizational policy, among others (10–12). In this sense, it is the capacity to face work demands, acting as a protective factor against stress (13). Thus, in the presence of high psychological demand with low control over work, a work situation can harm the individual’s health (10, 14).

Occupational stress affects workers’ health differently, given the diversity of work environments (1, 5). In this scenario, hospital workers have experiences that impact their health in different ways, including changes in their body composition (15, 16). Body composition encompasses the assessment of body parameters divided into fat-free mass, adipose tissue, body cell mass, total body water and extracellular water (17–19).

Adequate body composition is essential for an individual’s health because excess adipose tissue favors metabolic disorders and, consequently, contributes to the emergence and/or worsening of chronic diseases—cardiovascular diseases, diabetes mellitus, high blood pressure, etc. Furthermore, assessing body composition is important to obtain information about the nutritional status of individuals, and the functional capacity of the human body, in addition to offering information necessary for intervention measures, among others (19–21).

Occupational stress impacts lifestyle levels, represented by low frequency or absence of physical activity, alcohol and tobacco consumption, failure to prepare one’s own meals (22, 23), in addition to exposure to high food consumption, whether in volume and/or caloric density (24, 25). Thus, workers immersed in a stressful work environment are at risk of developing overweight and obesity. Therefore, body composition is a significant aspect of the study of stress because overweight workers have a higher risk of occupational injuries (26), reduced work capacity concerning the physical demands of the job (27, 28), decreased productivity or increased absence due to illness (16, 29). Still, it is especially necessary to evaluate development among hospital workers, as they are immersed in environments with precarious working conditions such as long working hours, many hours standing, shift work, among others.

There is a gap in the scientific literature that concerns the health of hospital workers when addressing the body composition of this group, as they basically use indicators such as the Body Mass Index (BMI), Waist Circumference (WC) and, to a lesser extent, the percentage of body fat, assessed through the sum of skinfolds. However, the use of anthropometry for such an assessment is limiting because it is a method in which accuracy and precision depend on experienced and trained evaluators, the use of appropriate and properly calibrated equipment, in addition to the regression models used in the formulas specific to each population, which makes it necessary to adopt specific and more complex parameters, among other factors (19, 21, 30). On the other hand, the gold standard methods for assessing body composition are magnetic resonance imaging and computed tomography, which are expensive and unavailable in most occupational medicine services, especially in continental and developing countries, such as Brazil. A good alternative is the use of Bioelectrical Impedance Analysis (BIA), which has a high correlation with anthropometric methods and techniques considered the gold standard, in addition to having a relatively low cost (19, 31). Given this context, this study sought to analyze the influence of occupational stress on hospital workers’ body composition (using BIA, BMI and WC) after one year of follow-up.

## 2 Materials and methods

### 2.1 Study design and sampling

The Ethics and Research Committee of the School of Nutrition at the Federal University of Bahia approved this study (CAAE approval number 4,316,252). The procedures followed the guidelines established by the Declaration of Helsinki and all workers who agreed to participate in the study signed an informed consent form.

This prospective cohort study included 218 workers from one of the leading private hospitals in the municipality of Santo Antônio de Jesus, Recôncavo da Bahia region, Northeast Brazil, with secondary and tertiary levels of care (32, 33). This municipality has an estimated population of 103.055 thousand inhabitants (34).

Sampling was non-probability and was carried out for convenience by inviting all the hospital workers. Of the 302 workers who began the study, 218 completed both interviews, totaling a definitive loss of 27.81% (*n* = 84). During follow-up, the loss occurred due to dismissal (*n* = 66), sick leave (*n* = 8), pregnancy (*n* = 4), vacation (*n* = 4) and withdrawal from participating in the study (*n* = 2). Data from the sample was used to calculate the sampling power, considering that specific information about the variability of exposure and outcome in a similar population was unknown and/or was not identified. Using sample data, we sought to estimate parameters necessary for calculating power, allowing a more adaptive and realistic approach given the initial uncertainty (35). Thus, this sample size (218 individuals) has power ranging from 91.9 to 99.9% to detect an unbiased association between occupational stress (in its different domains) and body composition (BMI, WC, FFM and CG) after a year of follow-up in the study population. The sample power calculations (1-∂) were based on the 5% significance level and two-tailed tests, indicating that this sample size is sufficient to make unbiased estimates of the parameters of the population under study (36).

The inclusion criteria adopted were being of legal age (≥ 18 years in Brazil), carrying out work activities (regardless of the job position), the possibility of carrying out anthropometric measurements and being able to carry out bioelectrical impedance analysis, in addition to women who are not pregnant or in the postpartum period. In turn, the exclusion criteria were no longer being a worker at the hospital under study, becoming pregnant, expressing the desire to leave the research, and being away from the work environment for reasons such as vacation or sick leave (37).

All measurements were collected at two follow-up moments: between May and October 2019, to form the study baseline; and between October and December 2020, for follow-up with the same workers, instruments, techniques and interviewers.

### 2.2 Study variables

#### 2.2.1 Outcome: body composition

Body composition was analyzed in two ways: the use of proxy and Bioelectrical Impedance Analysis (BIA). The proxies used were Body Mass Index (BMI) and Waist Circumference (WC). Anthropometric weight and height measurements were taken to calculate BMI later. A portable digital scale with bioimpedance on a platform was used to measure weight, where the workers were wearing light clothing, were barefoot, and were without adornments or any object that could interfere with the measurement. Height was measured with the worker in an upright posture, feet together and heels against the wall, using a portable stadiometer. The BMI was calculated using measurements of weight and height (weight–kg/height m^2^) (38). For inclusion in the model, the variable was used in a continuous and time-varying manner.

The WC was measured with a flexible and inelastic measuring tape. The men were asked to remove their blouses and the women to lift them at the waist. The workers stood upright, with their abdomen relaxed, arms extended and weight equally distributed between their legs, with their feet close and parallel. The measurement was taken at the end of expiration, at the midpoint marked between the last rib and the iliac crest, which was located and marked previously (39). For inclusion in the model, the variable was used in a continuous and time-varying manner.

Body composition was assessed using a tetrapolar bioelectrical impedance device (Biodynamics^®^), according to the protocol described by Kyle et al. (40): fasting for 4 h and no alcohol consumption in the 48 h before the exam; empty bladder before measurement; no physical exercise in the last 48 h; skin at room temperature and without visible lesions; the same side of the body was measured and with a minimum distance of 5 cm between the electrodes; the electrodes were cleaned with alcohol; arms separated from the trunk by around 30° and legs separated by around 45°; supine position for 5 to 10 min. There was no contact with the metal structure of the stretcher and in a neutral environment (without strong electric or magnetic fields). From the information provided by the BIA, we used Fat Free Mass (FFM) and Body Fat (BF) data. For inclusion in the model, both variables were used in a continuous and time-varying manner.

#### 2.2.2 Main exposure variable: occupational stress

The primary exposure adopted in the present study was the perception of occupational stress, assessed with the adapted and reduced version of the Job Content Questionnaire (JCQ), validated for the Brazilian population by Alves et al. (10) (Supplementary Table S1).

The JCQ was an instrument created by Robert Karasek to assess social relationships in the workplace that generated stress and impacted health outcomes. Originally, the instrument contained 49 questions distributed between the “Demand” and “Control” dimensions (10). Years later, in Sweden, Töres Theorell adapted the original instrument by reducing it to 11 questions and calling it the Job Stress Scale (JSS) distributed as follows: five questions to assess Demand and six questions to assess Control (10).

At the National School of Public Health in Rio de Janeiro—Brazil, Alves et al. (10), after obtaining the rights, adapted the scale once reduced by Töres Theorell in Sweden into Brazilian Portuguese. The adaptation has response options presented on a Likert scale (1–4 items), varying between “often” and “never/almost never” for the Demand and Control dimensions. With the adaptation process, Brazilian researchers obtained two reliability components of the scale, which were the intraclass correlation coefficients for the Demand (0.88) and Control (0.87) dimensions and the Cronbach’s alpha of 0.79 (Demand) and 0.67 (Control) also for both dimensions (10).

Workers were asked about both dimensions—Demand and Control—at both data collection times for this research. Given that the concepts of these dimensions are theoretically distinct (41, 42) and to achieve the objective of this study, we used information from the two dimensions separately (10, 43).

For its categorization, the first step was to construct the “Demand” and “Control” dimensions, which was done by adding up the questions present in the JSS. Then, to classify the worker as high demand (risk category) and low demand (reference category), and high control (reference category) and low control (risk category), we sum the scores above or below the value from the median (44).

#### 2.2.3 Covariates

The research form was prepared for the present study and completed by previously trained interviewers. Thus, the variables were grouped into: (a) work characteristics (job position: administrative and health professionals; working time in the position in full years: <1 year to 3 years, 4 to 5 years and ≥ 6 years; working hours weekly working hours: ≤44 h and ≥ 45 h (45); other employment: yes and no; work shift: shift, day and night; type of contract: permanent/contracted and others); (b) biological characteristics (biological sex: man and woman; reported race/color: white and non-white; age in years: 18 to 30 years, 31 to 43 years and 44 to 61 years); (c) sociodemographic characteristics (education: elementary/high school and higher education/specialization; family income in minimum wages: 1 to <3 and ≥ 3; number of dependents: 1 to 4 and ≥ 5); (d) lifestyle (smoking: yes and no; alcohol consumption: yes and no; hours of sleep: <7 h and ≥ 7 h; number of meals per day: 1 to 5 and ≥ 6; physical activity: low, moderate and high).

The physical activity variable was assessed with the reduced version of the International Physical Activity Questionnaire, validated for the Brazilian population (46). The workers were asked about the levels of activities (physical exercise, domestic, leisure and commuting activities) carried out seven days before data collection in the two study periods and considering the frequency and duration criteria. Finally, for this study, workers were classified as having low, moderate and high levels of physical activity, if they had Metabolic Equivalents (MET) <600/min/week, 600 to 3,000 MET/min/week and ≥ 3,000 MET/week minutes/week, respectively (47).

### 2.3 Statistical analysis

Statistical analyses were carried out in three main steps: (i) Descriptive analysis of sample characteristics: categorical variables were summarized by absolute (n) and relative (%) frequencies. Prevalences were estimated by demand and control categories (high or low) in each period. Quantitative variables were summarized using mean and standard deviation (SD), after applying the Shapiro–Wilk test to assess normality. The mean values of weight, BMI, WC, FFM and BF according to high or low demand and control in each period were compared using the Student’s t-test for equal or unequal variances. In the analyses, a significance of 5% was adopted (48).

(ii) Bivariate analysis (assessment of the association of outcomes with the main exposure and covariates): comparing outcome variables and covariates of interest according to the exposure variable was performed to select candidate variables for the multivariate model. Those associated with exposure and outcome, expressed by a change <20% in the *p*-value compared to that measured in the raw model, were considered potential confounders and included in the multivariate models using the Linear Mixed Effects Model analysis (48, 49). In addition to statistical criteria, the theoretical framework was considered when choosing the final model.

(iii) Multivariate analysis: to evaluate the influence of occupational stress (variant over time and used in a categorized way: demand and control) on the variation in BMI, WC, FFM and BF over time, we constructed Linear Mixed Effects Models (LMEM). Such analysis is pertinent when measurements repeated over time are obtained from the same individual, as it considers the correlation between measurements at each moment in time, as well as the variability between and within individuals over time, considering the temporal variable in the relationship between exposure and outcome (50, 51). To evaluate the desired relationship, a model was built for each outcome (anthropometric data included in its continuous and longitudinal form) as a function of the primary exposure variable (demand and control, included simultaneously in the models). The variables selected in step ii were included in the model as adjustment variables. For the present study, the correlation matrix chosen was auto-regressive, given that time had an equal space to obtain the measurements and that the data were not unbalanced (52).

The model’s adjusted data were evaluated based on the Akaike Information Criterion (AIC), where the lower the value, the better the model fit (53) and also by the likelihood ratio test. All analyses were performed using STATA^®^ for MAC Version 17.0 statistical software (StataCorp, College Station).

## 3 Results

At baseline, the median value for demand was 13 (IQR: 5–20) and for control, 15 (IQR: 8–24). At the first stage of the study, we identified that 55.96% (*n* = 122) of workers had high work demand and 25.22% (*n* = 55) had low control. Among those who had high demand and low control at the beginning of the study, the majority were women (71.31% vs. 70.91%), of race/skin color referred to as non-white (86.06% vs. 87. 27%), with primary/high school education (56.55% vs. 60%), income from 1 to <3 minimum wages (74.59% vs. 70.91%), with 1 to 4 dependents in the family (90 0.98% vs. 89.09%), with less than 7 h of sleep/day (59.83% vs. 61.82%), had 1 to 5 meals/day (88.52% vs. 83. 64%), without another job (81.14% vs. 76.36%), worked during the day shift (64.75% vs. 70.91%) and with other types of employment relationships other than those governed by labor legislation of the country (96.73% vs. 90.91%) (Table 1).

**Table 1:** Sociodemographic characteristics of workers at baseline, according to high or low presence of demand and control.

Variables	Categories	Demand		Control	
		High demand *n* = 122 (%)	Low demand, *n* = 96 (%)	High control *n* = 163 (%)	Low control, *n* = 55 (%)
Sex	Men	35 (28.68)	19 (19.79)	38 (23.31)	16 (29.09)
	Women	87 (71.31)	77 (80.21)	125 (76.69)	39 (70.91)
Age in years	18 to 30 years old	49 (40.16)	40 (41.67)	68 (41.72)	21 (38.18)
	31 to 43 years old	60 (49.18)	48 (50)	80 (49.08)	28 (50.91)
	44 to 61 years old	13 (10.65)	8 (8.33)	15 (9.20)	6 (10.91)
Race/color	White	17 (13.93)	11 (11.46)	21 (12.88)	7 (12.73)
	Not white	105 (86.06)	85 (88.54)	142 (87.12)	48 (87.27)
Education	Elementary/high school	69 (56.55)	50 (52.08)	86 (52.76)	33 (60)
	Higher education/specialization	53 (43.44)	46 (47.92)	77 (47.24)	22 (40)
Family income	1 to <3 minimum wages	91 (74.59)	69 (71.88)	121 (74.23)	39 (70.91)
	≥3 minimum wages	31 (25.40)	27 (28.13)	42 (25.77)	16 (29.09)
Number of dependents	1 to 4 dependents	111 (90.98)	85 (88.54)	147 (90.18)	49 (89.09)
	≥5 dependents	11 (9.02)	11 (11.46)	16 (9.82)	6 (10.91)
Smoking	Yes	1 (0.81)	1 (1.04)	2 (1.23)	0 (0)
	No	121 (99.17)	95 (98.96)	161 (98.78)	55 (100)
Alcoholism	Yes	71 (58.19)	42 (43.75)	84 (51.53)	29 (52.73)
	No	51 (41.80)	54 (56.25)	79 (48.47)	26 (47.27)
Hours of sleep	<7 h	73 (59.83)	60 (62.50)	99 (60.74)	34 (61.82)
	≥7 h	49 (40.16)	36 (37.50)	64 (39.26)	21 (38.18)
Number of meals	1 to 5 meals/day	108 (88.52)	86 (89.58)	148 (90.80)	46 (83.64)
	≥6 meals/day	14 (11.47)	10 (10.42)	15 (9.20)	9 (16.36)
Physical activity	Low	51 (41.80)	34 (35.42)	67 (41.10)	18 (32.73)
	Moderate	54 (44.26)	42 (43.75)	69 (42.33)	27 (49.09)
	High	17 (13.93)	20 (20.83)	27 (16.56)	10 (18.18)
Job role	Administrative	64 (52.45)	63 (65.63)	94 (57.67)	33 (60)
	Health professionals	58 (47.54)	33 (34.38)	69 (42.33)	22 (40)
Working time in position	<1 to 3 years	49 (40.16)	45 (46.88)	80 (49.08)	14 (25.45)
	4 to 5 years	33 (27.04)	28 (29.17)	47 (28.83)	14 (25.45)
	≥6 years	40 (32.78)	23 (23.96)	36 (22.09)	27 (49.09)
Working hours	≤44 h/week	90 (73.8)	78 (81.2)	126 (77.3)	42 (76.4)
	≥45 h/week	32 (26.2)	18 (18.8)	37 (22.7)	13 (23.6)
Another job	Yes	23 (18.85)	17 (17.71)	27 (16.56)	13 (23.64)
	No	99 (81.14)	79 (82.29)	136 (83.44)	42 (76.36)
Work shift	Duty	40 (32.78)	28 (29.17)	54 (33.13)	14 (25.45)
	Daytime	79 (64.75)	67 (69.79)	107 (65.64)	39 (70.91)
	Nocturnal	3 (2.45)	1 (1.04)	2 (1.23)	2 (3.64)
Type of contract	Effective/contracted	4 (3.27)	7 (7.29)	6 (3.68)	5 (9.09)
	Others	118 (96.73)	89 (92.71)	157 (96.32)	50 (90.91)

Santo Antônio de Jesus, Bahia, Brazil, 2019–2020.

After 12 months of follow-up, the median value for demand continued as 13 (IQR: 5–25) and for control, it increased to 16 (IQR: 9–23). In this second moment of the study, 62.38% (*n* = 136) of workers showed high demand and 45.87% (*n* = 100) low control. Among workers with high demand and low control, the majority remained women (71.32% vs. 73%), of race/skin color referred to as non-white (86.03% vs. 90%), with an income of 1 to <3 minimum wages (64.71% vs. 65%), with 1 to 4 dependents in the family (91.91% vs. 89%), had 1 to 5 meals/day (88.24% vs. 90%), had no other employment relationship (80.15% vs. 76%) and had other forms of employment other than the consolidation of Brazilian labor laws (96.32% vs. 91%) (Table 2).

**Table 2:** Sociodemographic characteristics of workers after 12 months of follow-up, according to high or low presence of demand and control.

Variables	Categories	Demand		Control	
		High demand *n* = 136 (%)	Low demand *n* = 82 (%)	High control *n* = 118 (%)	Low control *n* = 100 (%)
Sex	Men	39 (28.68)	15 (18.29)	27 (22.88)	27 (27)
	Women	97 (71.32)	67 (81.71)	91 (77.12)	73 (73)
Age in years	18 to 30 years old	49 (36.03)	33 (40.24)	45 (38.14)	37 (37)
	31 to 43 years old	73 (53.68)	41 (50)	59 (50)	55 (55)
	44 to 61 years old	14 (10.29)	8 (9.76)	14 (11.86)	8 (8)
Race/color	White	19 (13.97)	9 (10.98)	18 (15.25)	10 (10)
	Not white	117 (86.03)	73 (89.02)	100 (84.75)	90 (90)
Education	Elementary/high school	69 (50.74)	50 (60.98)	65 (55.08)	54 (54)
	Higher education/specialization	67 (49.26)	32 (39.02)	53 (44.92)	46 (46)
Family income	1 to <3 minimum wages	88 (64.71)	57 (69.51)	80 (67.80)	65 (65)
	≥3 minimum wages	48 (35.29)	25 (30.49)	38 (32.20)	35 (35)
Number of dependents	1 to 4 dependents	125 (91.91)	73 (89.02)	109 (92.37)	89 (89)
	≥5 dependents	11 (8.09)	9 (10.98)	9 (7.63)	11 (11)
Smoking	Yes	3 (2.21)	1 (1.22)	3 (2.54)	1 (1)
	No	133 (97.79)	81 (98.78)	115 (97.46)	99 (99)
Alcoholism	Yes	91 (66.91)	40 (48.78)	74 (62.71)	57 (57)
	No	45 (33.09)	42 (51.22)	44 (37.29)	43 (43)
Hours of sleep	<7 h	72 (52.49)	42 (51.22)	67 (56.78)	47 (47)
	≥7 h	64 (47.06)	40 (48.78)	51 (43.22)	53 (53)
Number of meals	1 to 5 meals/day	120 (88.24)	74 (90.24)	104 (88.14)	90 (90)
	≥6 meals/day	16 (11.76)	8 (9.76)	14 (11.86)	10 (10)
Physical activity	Low	45 (33.09)	16 (19.51)	37 (31.36)	24 (24)
	Moderate	69 (50.74)	57 (69.51)	68 (57.63)	58 (58)
	High	22 (16.18)	9 (10.98)	13 (11.02)	18 (18)
Job role	Administrative	74 (54.41)	53 (64.63)	68 (57.63)	59 (59)
	Health professionals	62 (45.59)	29 (35.37)	50 (42.37)	41 (41)
Working time in position	<1 to 3 years	58 (42.65)	36 (43.90)	55 (46.61)	39 (39)
	4 to 5 years	39 (28.68)	22 (26.83)	34 (28.81)	27 (27)
	≥6 years	39 (28.68)	24 (29.27)	29 (24.58)	34 (34)
Working hours	≤44 h/week	94 (69.1)	58 (70.7)	86 (72.9)	66 (66)
	≥45 h/week	42 (30.9)	24 (29.3)	32 (27.1)	34 (34)
Another job	Yes	27 (19.85)	14 (17.07)	17 (14.41)	24 (24)
	No	109 (80.15)	68 (82.93)	101 (85.59)	76 (76)
Work shift	Duty	53 (38.97)	33 (40.24)	45 (38.14)	41 (41)
	Daytime	80 (58.82)	49 (59.76)	72 (61.02)	57 (57)
	Nocturnal	3 (2.21)	0 (0)	1 (0.85)	2 (2)
Type of contract	Effective/contracted	5 (3.68)	6 (7.32)	2 (1.69)	9 (9)
	Others	131 (96.32)	76 (92.68)	116 (98.31)	91 (91)

Santo Antônio de Jesus, Bahia, Brazil, 2019–2020.

Evaluating the average of the outcomes, workers in high demand had the highest indicators in both periods. At baseline, only BMI showed a statistically significant difference between workers with high and low demand (*p* = 0.024), whereas, after follow-up, only WC (*p* = 0.035) and FFM (*p* = 0.047) showed a difference statistically significant between workers with high and low demand (Table 3).

**Table 3:** Average values of body composition indicators according to demand and control at baseline and after follow-up, in hospital workers in Santo Antônio de Jesus, Bahia, Brazil, 2019–2020.

Variables	Baseline			Follow-up		
	High demand (*n* = 122)	Low demand (*n* = 96)	*p*-value	High demand (*n* = 136)	Low demand (*n* = 82)	*p*-value
	Mean (SD)	Mean (SD)		Mean (SD)	Mean (SD)	
Weight (kg)	70.61 (14.94)	67.48 (12.57)	0.102	72.19 (15.44)	68.59 (12.22)	0.074
BMI (kg/m^2^)	26.14 (4.83)	24.72 (4.24)	**0.024**	26.45 (4.79)	25.54 (4.63)	0.174
WC (cm)	85.03 (11.86)	83.26 (10.33)	0.250	88.98 (12.38)	85.54 (10.20)	**0.035**
FFM (kg)	49.99 (9.80)	47.78 (8.41)	0.090	50.47 (10.09)	47.85 (8.01)	**0.047**
BF (kg)	20.29 (8.35)	19.23 (7.43)	0.348	21.86 (8.73)	20.78 (7.39)	0.335

Variables	Baseline		*p*-value	Follow-up		*p*-value
	High control (*n* = 163)	Low control (*n* = 55)		High control (*n* = 118)	Low control (*n* = 100)	
	Mean (SD)	Mean (SD)		Mean (SD)	Mean (SD)	
Weight (kg)	68.74 (14.25)	70.69 (13.24)	0.372	68.55 (13.80)	73.55 (14.68)	**0.010**
BMI (kg/m^2^)	25.22 (4.50)	26.38 (4.93)	0.110	25.38 (4.65)	26.97 (4.74)	**0.013**
WC (cm)	84.03 (11.09)	84.90 (11.68)	0.618	86.38 (11.61)	89.23 (11.68)	0.073
FFM (kg)	48.77 (9.41)	49.83 (8.88)	0.480	47.99 (9.05)	51.24 (9.60)	**0.012**
BF (kg)	19.57 (7.53)	20.61 (9.20)	0.424	20.51 (7.80)	22.54 (8.66)	0.072

Subtitle: BMI: Body Mass Index; WC: Waist Circumference; FFM: Fat Free Mass; BF: Body Fat. Values in bold refer to those that were statistically significant in the Student’s *t*-test.

Regarding workers with high and low control at baseline, higher mean values for weight, BMI, WC, FFM and BF were identified in workers with low control. However, no statistically significant difference was observed for the outcomes evaluated. After follow-up, all mean values of the outcome variables were higher for workers with low control, with the difference being statistically significant for weight (*p* = 0.010), BMI (*p* = 0.013) and FFM (*p* = 0.012) (Table 3).

Table 4 presents the raw and adjusted LMEM results for the relationship between stress (assessed through the dimensions of demand and control) and adiposity indicators. After adjusting for age, weekly working hours, alcohol consumption, physical activity, having another job and education, we observed that individuals classified as high demand had an increase of 0.979 kg/m^2^ (*p* = 0.023) in mean BMI after the follow-up period, when compared to those classified as low demand; on the other hand, individuals classified in the low control category had a decrease of −1,180 kg/m^2^ (*p* = 0.008) in the mean of this indicator after follow-up, when compared to those classified as high control (Table 4).

**Table 4:** Linear mixed effects model for the relationship between occupational stress and body composition at 12 months of follow-up.

Fixed effects	Body mass index (Kg/m^2^)					
	Raw model			Final model*		
	Estimate	Standard error	*p*-value	Estimate	Standard error	*p*-value
Intercept	26.052	0.456	0.000	22.472	2.721	0.000
High demand^♣^	1.197	0.449	0.008	0.979	0.432	0.023
Low control^♣♣^	−1.466	0.461	0.001	−1.180	0.443	0.008
Random effects	Minimum	Estimate	Maximum	Minimum	Estimate	Maximum
Residue	18.628	21.282	24.315	16.596	18.990	21.730
Log Restricted-Likelihood: −1284.1042				−1257.8381		
AIC	2576.208			2543.676		

Fixed effects	Waist circumference (cm)					
	Raw model			Final model**		
	Estimate	Standard error	*p*-value	Estimate	Standard error	*p*-value
Intercept	85.783	1.731	0.000	63.862	6.121	0.000
High demand^♣^	2.636	1.111	0.018	1.915	1.043	0.066
Low control^♣♣^	−2.127	1.162	0.067	−1.661	1.094	0.129
Random effects	Minimum	Estimate	Maximum	Minimum	Estimate	Maximum
Residue	113.469	129.657	148.154	98.588	112.758	128.964
Log Restricted-Likelihood: −1676.2542				−1641.09		
AIC	3362.508			3304.18		

Fixed effects	Fat free mass (kg)					
	Raw model			Final model^†^		
	Estimate	Standard error	*p*-value	Estimate	Standard error	*p*-value
Intercept	49.311	0.932	0.000	55.328	1.993	0.000
High demand^♣^	2.423	0.915	0.008	0.957	0.706	0.176
Low control^♣♣^	−2.306	0.939	0.014	−1.396	0.724	0.054
Random effects	Minimum	Estimate	Maximum	Minimum	Estimate	Maximum
Residue	74.257	85.039	97.386	43.435	49.774	57.038
Log Restricted-Likelihood: −1529.342				−1415.6136		
AIC	3066.684			2847.227		

Fixed effects	Body fat (kg)					
	Raw model			Final model^&^		
	Estimate	Standard error	*p*-value	Estimate	Standard error	*p*-value
Intercept	21.106	0.936	0.000	6.760	3.971	0.089
High demand^♣^	1.124	0.802	0.161	1.448	0.719	0.044
Low control^♣♣^	−1.744	0.834	0.037	−1.782	0.729	0.015
Random effects	Minimum	Estimate	Maximum	Minimum	Estimate	Maximum
Residue	56.976	65.259	74.746	43.813	50.249	57.630
Log Restricted-Likelihood: −1474.4161				−1412.0226		
AIC	2958.832			2852.045		

Santo Antônio de Jesus, Bahia, Brazil, 2019–2020. *Adjusted by age, weekly working hours, alcohol consumption, physical activity, having another job and education. **Adjusted by age, weekly working hours, number of meals/day, having another job, employment contract and work shift. †Adjusted for gender, physical activity, weekly working hours, education and having another job. &Adjusted by age, number of meals/day, working time, weekly working hours, gender, having another job, smoking, work shift. ♣Low demand (reference category). ♣♣High control (reference category).

For WC, only high demand (estimate: 2.636; *p* = 0.018) presented a statistically significant result in the crude model. However, when adjusting for age, weekly working hours, number of meals/day, having another job, contract work and work shift, this significance did not remain (*p* = 0.066).

For FFM, in the crude model, high demand (estimate: 2.423; *p* = 0.008) was significant, but the significance was not maintained when adjusting for covariates. As for control, we observed that workers classified as low control had a reduction of −1,396 kg (*p* = 0.054) in the mean FFM after follow-up, compared to those classified as high control (Table 4).

When evaluating the BF outcome, we observed that, in the adjusted model, individuals classified as high demand showed an increase of 1,448 kg (*p* = 0.044) in mean BF after the follow-up period, compared to those with low demand. For control, workers classified as low control showed a decrease of −1,782 kg (*p* = 0.015) in mean BF after follow-up, compared to workers with high control (Table 4).

The constructed models were well adjusted to the data, according to the AIC and Log Restricted-Likelihood criteria identified, considering that there was a reduction in these indicators in the final models when compared to the raw models (Table 4).

## 4 Discussion

This investigation suggests that high demand at work contributed to the increase in average BMI and body fat values, while low control at work reduced average BMI and BF values after one year of follow-up. Furthermore, low control at work influenced the average reduction in BMI, BF and FFM.

Assessing and monitoring workers’ body composition is essential to identify possible nutritional changes, contributing to improving health and performance of work activities (8, 54, 55). Despite the importance of the topic, we observe a rare scenario of studies in the literature that investigate longitudinal information, incorporating temporal variations between occupational stress and body composition in hospital workers.

Investigating the consequences of exposure to occupational stress, most studies adopt cross-sectional designs, which are unable to identify changes that occur in the work environment and that may impact health, as well as not being able to detect the influence of working conditions work on the health status of workers (56). Furthermore, they limit themselves to using variables such as weight, BMI and WC and do not use data from tests such as bioimpedance (23). Longitudinal studies are relevant because they allow the identification of variations of the same variable over time and consider the observations of a subject with himself and him with another subject over time. As a result, estimates become more precise, and the test is more sensitive to capturing significant differences (51, 57).

This investigation provides longitudinal information that suggests that high demand and low control influence BMI, therefore, BMI may be a consequence of working conditions. In this sense, this result corroborates previous findings where Bean et al. (42) found in a cohort in South Australia with 450 employees, that an increase of 1 unit in discretionary skill was associated with a decrease of 0.168 kg/m^2^ in the crude analysis of BMI (*p* = 0.005). Fujishiro et al. (58), in a cohort of nurses from the Nurses’ Health Study II, when associating changes in work tension and changes in BMI, identified that those who experienced a decrease (b = 0.063; *p* = 0.03) or increase (b = 0.077; *p* = 0.01) of work stress had greater BMI gain than those without exposure to work stress at any time.

At this juncture, working conditions can trigger stress and thus contribute to harmful outcomes for workers’ health, such as changes in the sleep–wake cycle, overweight and obesity (23, 25, 59, 60). Occupational stress is a worldwide public health problem that can influence body weight in several ways, such as, for example, favoring increased consumption of foods, that are primarily hyper-palatable and ultra-processed, and immersion in work activities. In everyday life, the individual does not notice the change in consumption patterns and the unhealthy increase in weight (24, 61, 62).

Eating habits can be altered by stressful situations (63), especially ego-threatening stressors (e.g., situations where there is “fear of failure”) and interpersonal stressors (e.g., ostracism, arguments) (64–66). Thus, when a worker is exposed to stress, his or her hypothalamus stimulates the pituitary gland to release corticotropin-releasing factor, leading to the secretion of adrenocorticotropic hormone in the anterior pituitary gland. This hormone then stimulates the adrenal glands to increase the rate of cortisol synthesis and release (67). High levels of cortisol, which also increase the sensitivity of the brain’s reward system, can lead to greater intake of hyperpalatable foods (24, 61, 62), increased consumption of saturated fat and sugar (68, 69), among others.

It is known that stress can be experienced from different dimensions of the environment due to physiological, psychological and social stressors (70). Given the complex social structure and the fact that control over the stressor does not always depend on it, the effects on appetite and weight may appear differently. Thus, recognizing the causes of stress is paramount in trying to reverse the problems (61). It is also essential to understand that positive energy balance, that is, the amount of calories ingested is greater than those expended, is not solely and exclusively in the physiological scope, but also in the environmental, social, cultural and genetic sphere (71).

The dynamics of the work carried out in hospitals—due to the uninterrupted operation, a large number of professions, the work shared between different occupations, the differences inherent to each work activity, the hierarchical rigidity and the undersizing of human resources—is favorable for trigger occupational stress (72–74). This population has stressful work dynamics with risk factors for increased weight and body fat, until overweight and obesity sets in Kołcz et al. (23). Among health-care professionals, for example, long working hours and shift work are observed (75–78), as well as limitations in the practice of physical activity (79, 80), reduction in the quality of food intake (23, 68, 79, 80), and undesirable impacts on sleep (81, 82).

Therefore, the literature is consistent in identifying that overweight and obesity are highly prevalent among hospital workers, especially health-care professionals (83–86). Furthermore, it is known that, despite being distinct, nutritional status and occupational stress are related (87, 88). Thus, in the presence of stress, nutritional status can be affected and, in this study, the dimensions of occupational stress assessed with the JSS demonstrated an influence on the increase in BMI. This finding was similar to those of Fujishiro et al. (58) in a longitudinal study with nurses, in which they identified that those with tension at work at both times increased their BMI compared to nurses who did not experience tension at work. A Swiss study of manual and administrative workers also identified that control and social stressors were predictors of BMI during study follow-up (89). Kivimäki et al. (90), in a cohort of workers from 20 Public Service departments in the Whitehall II study in London, identified that among men, high tension and low control at work in the highest BMI quintile (>27 kg/m^2^), were associated with later weight gain, but no corresponding interaction was observed among women.

In epidemiological studies evaluating the anthropometric status of individuals, only the BMI indicator is used, which, despite having the advantages of low cost, easy performance and serving as an indicator for more sensitive assessments, only considers weight regarding the individual’s height. In an individualized context, this is not the best option, as it does not distinguish body composition and distribution nor does it consider other aspects of obesity, such as genetic, metabolic, physiological or psychological factors. In other words, it does not diagnose, it only serves as an indicator for a more sensitive assessment and, in population assessments, it is the best tool available to date (21, 91). Thus, among the most accurate and reliable alternatives, the use of electrical bioimpedance is a good choice, as it is low cost, non-invasive, has good reproducibility, and there is the possibility of working with portable and easy-to-use equipment (20, 31, 37). However, despite these advantages, its use is still limited in the occupational medicine services of organizations.

This study identified a significant association between high demand and FFM in the raw model, high demand and BF in the adjusted model (*p* = 0.044) and low control with FFM and BF. The “Demand” dimension present in the instrument refers to how much the individual works, that is, it observes issues related to the development of their work activities, such as volume, pace, workload, etc. (43, 44, 60), while the “Control” dimension involves making decisions about one’s work and how much they develop creativity, learning from new things at work, carrying out multiple tasks, among others (43, 44, 60).

Thus, the findings can be explained by the characteristics that such dimensions represent in hospital work, where it is observed that workers have, for example, a large volume of work, long working hours, undersized staff, decision-making needs to take place in conjunction with other professionals and multiple tasks are performed, issues that contribute to work overload, impact on lifestyle, favoring the accumulation of adipose tissue and reduction in lean mass.

Furthermore, the mechanisms underlying the reduction in mean BMI and BF values associated with low control at work after one year of follow-up may have been because, for high control, workers need, among other things, to be able to make decisions about their own work and be able to develop their creativity. During the pandemic, such autonomy was not possible, and there was a need to perform multiple tasks. Another contributing factor was that most of our sample were women who historically experienced double work shifts. That is, in societies such as Brazil, even after working in the hospital, they are largely responsible for domestic work (92). This intensified during the pandemic, especially for those who were health professionals, due to the assistance and support they needed to offer to their families. Therefore, this scenario impacts diet, physical activity practices, and, consequently, body composition.

As far as we could ascertain, this study is a pioneer study in such an assessment with hospital workers and with this methodological approach. We did not identify studies that tested associations between occupational stress and FFM or BF in hospital workers, but we identified in a cross-sectional analysis and using BIA in Piauí—Brazil, with university hospital workers that 49.1% of the 53 health professionals studied had altered BF% and 15.1 had altered skeletal muscle mass index (82). In India, a study with 1,150 individuals, including students and health professionals, observed, using BIA, higher average values of %BF regardless of age group, sex and life cycles (adults vs. older adult) (93).

Given the above, we reaffirm that checking the body composition of individuals is important to identify possible nutritional disorders, in addition to guiding nutritional behaviors (31). In the field of workers’ health, this assessment remains necessary, mainly due to the labor dynamics within organizations and the growing and permanent precariousness of imposed working conditions. Although overweight and obesity are not occupational diseases, the high levels observed in institutions cannot be ignored. These high prevalences present challenges not only at the individual level, but also for the institution (42), resulting in more accidents at work and sick days (8), impacting productivity and incurring more expensive health plans for employers (94, 95), increased rates of absenteeism and presenteeism (16, 95), among other problems.

At the same time, it is worth highlighting the importance of evaluating stressful factors according to the different groups of professionals allocated to hospitals, especially among health workers, as depending on the role performed, work dynamics change and so do the risks of unwanted changes in body composition (55, 83, 84, 86). Despite this importance, it was not possible to perform such stratification in our study due to the sample size, which becomes a challenge to be achieved in future investigations.

Longitudinal investigations commonly have limitations. Firstly, in this study that data collection, at the time of follow-up, took place in the context of a pandemic, something rare to happen and, even historically, this workspace is recognized as favorable to occupational stress, a pandemic state certainly reinforces the high levels of stress experienced by these workers. However, we reinforce that the data collection and conduct of the study took place in the same way as in the baseline, that is, without a state of public calamity.

A second limitation is possibly the study’s follow-up period (12 months), which may not have been sufficient to observe some changes in body composition, especially variations in adiposity. However, the data obtained is reliable, as it comes from research with a robust methodology, where monitoring and conduction were well defined. Even though it presents such a limitation, we reinforce that the evidence listed here is based on a cohort study design with careful logistics and adoption of statistical treatment that is more appropriate to the data structure and an epidemiological approach. Furthermore, although the findings corroborate many studies with a cross-sectional design, this confirms the importance and need to study the influence of occupational stress on changes in body composition in hospital workers over time. A third limitation is that in this study, information on the use of medications (e.g., systemic corticosteroids, antidepressants, etc.) was not collected as well as the lack of detailed information on how moch and what the participant consume in each meal.

## 5 Conclusion

The study results indicate that high demand and low control at work are risk factors for changes in body mass index, fat mass and fat-free mass in hospital workers. This study shows the importance and need for clinical and epidemiological assessments regarding the body composition of professionals working in hospitals, since high rates of overweight and obesity are triggers of chronic health problems such as dyslipidemia, diabetes mellitus and cardiovascular diseases, among others. Therefore, managers need, initially, to promote adequate working conditions and understand the need for periodic body composition assessments.

Furthermore, our findings encourage future research on body composition associated with the dimensions of occupational stress independently in this population, instead of using present or absent stress globally, especially from a longitudinal perspective. Finally, even though our findings are not capable of bringing recommendations for changes in policies, it is worth highlighting that even though occupational stress is seen as prevalent in hospital work, it is urgent to realize that the work environment is potentially modifiable at the individual, collective and/or organizational.

## Data Availability

The original contributions presented in the study are included in the article/Supplementary material, further inquiries can be directed to the corresponding author.

## Supplementary material

SUPPLEMENTARY TABLE S1 Job content questionnaire.
