# Supplementary material for: Occupational stress and body composition of hospital workers: a follow-up study
Source: Front Public Health. 2024 Oct 17;12:1459809. doi: 10.3389/fpubh.2024.1459809 (PMC11524868; doi:10.3389/fpubh.2024.1459809)
Supplement: SUPPLEMENTARY TABLE S1 — Job content questionnaire. [file Table_1.pdf]

OCCUPATIONAL STRESS AND BODY COMPOSITION OF HOSPITAL WORKERS: A FOLLOW-UP STUDY

Carlos Rodrigo Nascimento de Lira, Rita de Cássia Coelho de Almeida Akutsu, Lorene Gonçalves Coelho, Renata Puppin Zandonadi, Priscila Ribas de Farias Costa

Occupational stress questionnaire (baseline and follow-up) used in the research

Job Content Questionnaire	
<u>Answer options from A to K: Often; sometimes; rarely; never or almost never.</u>	
a) How often do you have to do your work tasks very quickly?	
b) How often do you have to work very quickly (i.e., produce a lot in a short time)?	
c) Does your job demand too much of you?	
d) Do you have enough time to complete all of your work tasks?	
e) Does your job often present contradictory or discordant demands?	
f) Do you have the opportunity to learn new things at work?	
g) Does your job require a lot of skill or specialized knowledge?	
h) Does your job require you to take initiative?	
i) In your job, do you have to repeat the same tasks over and over again?	
j) Can you choose HOW to do your job?	
k) Can you choose WHAT to do at work?	
<u>Response options L through Q: I totally agree; I agree more than I disagree; I disagree more than I agree; I totally disagree.</u>	
l) There is a calm and pleasant environment where I work.	
m) At work, we get along well with each other.	
n) I can count on the support of my coworkers.	
o) If I'm not having a good day, my coworkers understand.	
p) At work, I get along well with my bosses.	
q) I enjoy working with my coworkers.	
